# Artificial intelligence-assisted bronchoscopy for the diagnosis and treatment of pulmonary diseases: Advances and clinical practice

**DOI:** 10.1515/jtim-2026-0068

**Published:** 2026-10-23

**Authors:** Guoliang Zhang, Yidan Qing, Xianwen Sun, Yi Xiang, Min Zhou, Qingyun Li, Jieming Qu, Wei Chen

**Affiliations:** Department of Pulmonary and Critical Care Medicine, Ruijin Hospital, Shanghai Jiao Tong University School of Medicine, Shanghai, China; Shanghai Key Laboratory of Severe Respiratory Infection, Shanghai, China; Institute of Respiratory Diseases, Shanghai Jiao Tong University School of Medicine, Shanghai, China

## Introduction

Artificial intelligence (AI) is transforming bronchoscopy into a precise, data-driven diagnostic and therapeutic modality. This perspective article summarizes recent AI advances across the bronchoscopy workflow, including image recognition, navigation, pathological interpretation, intraoperative assistance, quality control, and robotic systems. Clinical evidence shows AI can automatically detect and risk-stratify CT lung nodules, improve complex lesion path planning *via* 3D reconstruction and virtual bronchoscopy, and enable rapid biopsy interpretation with real-time on-site evaluation. AI also standardizes procedures, optimizes anatomical coverage, and integrates with robotic platforms to expand clinical potential. However, challenges in model generalizability, regulatory approval, and workflow integration persist. In the future, fusing AI with augmented reality, multimodal data, and intelligent robotics promises a personalized bronchoscopy ecosystem, enhancing diagnostic accuracy, procedural efficiency, and patient outcomes in lung disease management.

## Artificial intelligence and its integration with bronchoscopy

AI focuses its core efforts on endowing machines with the ability to simulate and expand human intelligent behavior, which encompasses deep learning, natural language processing, image recognition, and *etc*. In the medical field, the application of AI has evolved from early-stage auxiliary diagnosis to advanced capabilities such as lesion segmentation, pathology image recognition, surgical navigation, and collaborative operation with surgical robots, which drives the transformation of medical paradigm from traditional experience-based medicine toward to AI empowered precision and personalized care.

Bronchoscopy, as one of the key techniques for the diagnosis and treatment of pulmonary diseases, has long been challenged by complex lesion anatomy and difficulties in target localization. With the continuous rise in lung cancer incidence and the increasing burden of chronic respiratory diseases, there is an urgent demand for diagnostic and therapeutic approaches that offer higher precision, greater efficiency, and enhanced visualization. Thus, AI is gradually being integrated into the bronchoscopy workflow from lesion detection, three-dimensional modeling, navigation path selection and pathological analysis, establishing a smart bronchoscopy paradigm. Figure 1 shows the highlights flowchart of clinical practice and exploration of AI-assisted bronchoscopy in pulmonary diseases diagnosis and treatment.

**Figure 1:**
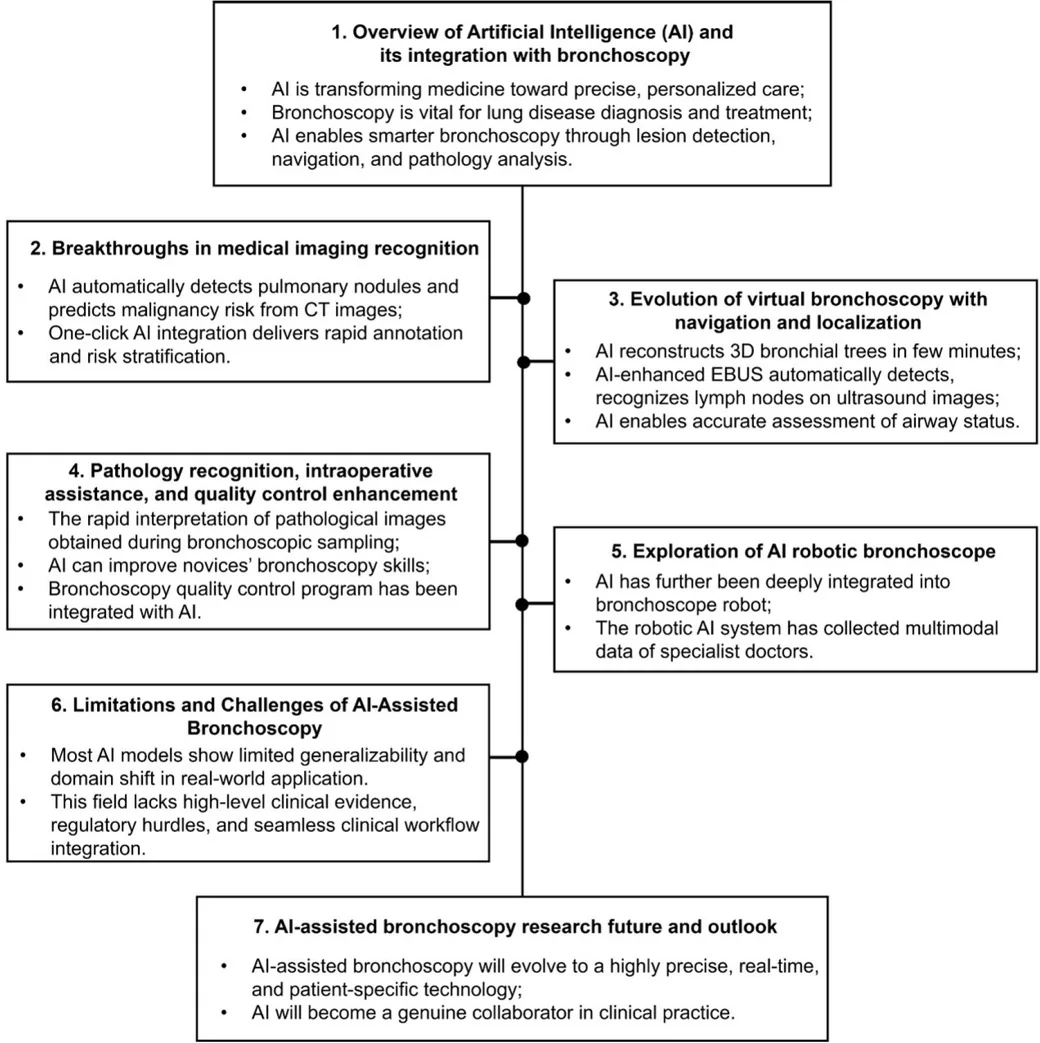
Highlights flowchart of clinical practice and exploration of artificial intelligence (AI)-assisted bronchoscopy in pulmonary diseases diagnosis and treatment.

## Breakthroughs in medical imaging recognition

One of the mature applications of AI at present is medical image recognition. AI can automatically detect nodule in CT images, analyze their size, density, margins, location, and other features, thereby outputting a probability score for benign versus malignant classification based on trained models. In clinical practice, a 6 mm part-solid nodule with a density of –400 HU, a certain proportion of solid components, and ill-defined margins can be identified as a high-risk lesion, prompting further diagnostic or therapeutic interventions.

Currently, several AI companies such as SenseTime and Lianyin Intelligent have integrated these models into hospital information system, allowing physicians to complete nodule annotation, and risk stratification with just one click on the system interface. This rapid-response capability has significantly improved outpatient efficiency, particularly in primary-level hospitals or settings with limited radiologist manpower, where AI systems have effectively replaced a portion of repetitive tasks.

## Evolution of virtual bronchoscopic navigation and localization

Traditionally, route planning relied heavily on physicians’ manual interpretation of radiologist-reconstructed virtual bronchial tree, which was tedious and time-consuming. In contrast, AI enables physicians to obtain a full 3D reconstruction in just few minutes by simply uploading CT images. For example, Fujifilm’s SYNAPSE 3D automatically integrates CT images to reconstruct spatial models of bronchi, vasculature, and target lesions. This enables physicians to determine bronchial accessibility and feasible puncture angle, which is valuable for distal, anatomically complex peripheral pulmonary lesions.^[1]^ Clinically, AI-assisted virtual bronchoscopic navigation (VBN) has demonstrated significant improvements in diagnostic performance. Studies report that VBN guidance increases the diagnostic yield of transbronchial needle aspiration to 70.9%–76.8% for mediastinal lymphadenopathy and achieves diagnostic accuracies of 65.4%–81.6% for peripheral pulmonary lesions, particularly for small nodes (1–2 cm) that are traditionally difficult to target.^[2]^

AI-enhanced EBUS automatically detects, recognizes, and localizes mediastinal lymph nodes on ultrasound images, improving diagnostic accuracy.^[2]^ It also guides biopsy decisions by integrating key imaging features (*e.g*., margins, echogenicity, and morphology) to distinguish benign from malignant nodes.^[3]^ Recent advances have further provided quantitative evidence supporting its clinical value. A deep learning framework applied to EBUS elastography achieved a diagnostic accuracy of 81.29% with an AUC of 0.8749, while reducing interpretation time from hours to seconds.^[4]^ In patients with post-operative airway fistulas, AI automatically measures the diameters of the proximal and distal fistula orifices as well as their spatial distance, facilitating customized stent selection and individualized placement with markedly success rates. In patients with atelectasis or bronchial stenosis, AI-based pathway analysis system assists physicians in precisely planning high-frequency electrocautery or airway dilation trajectories. AI is also widely applied in the quantitative assessment of chronic airway diseases such as asthma and chronic obstructive pulmonary disease (COPD). By objectively quantifying luminal area (LA), wall area (WA), and mean wall thickness (WT), AI enables accurate assessment of airway status, facilitating diagnosis, disease severity stratification, and evaluation of treatment.

## Pathology recognition, intraoperative assistance, and quality control enhancement

Another important application of AI lies in the rapid interpretation of pathological images obtained during bronchoscopic sampling. For example, pathologists upload histological slides to Fosun Pharma’s AI-ROSE system, which rapidly identifies cell types and staining patterns, provides preliminary benign/malignant judgments, and acts as a powerful initial screening tool for pathology labs.^[4]^ The large-language pathology model “RuiPath”, jointly developed by Ruijin Hospital Affiliated to Shanghai Jiao Tong University School of Medicine and Huawei, enables pre-identification of lesion areas in seconds per slide, which effectively shifts the pathologist’s workflow from manual microscopic search to interactively reviewing and confirming AI-generated results. In clinical implementation, advanced deep learning frameworks, such as the multi-scale fusion model M3-Net, have achieved an AUC of 0.83 in classifying pulmonary lesions, outperforming conventional models by up to 4%.^[1]^ These findings underscore the ability of AI to enhance diagnostic accuracy while substantially reducing interpretation time, thereby improving workflow efficiency and consistency in bronchoscopic pathology assessment.

Furthermore, AI can also improve novices’ bronchoscopy skills. In a previous study, 101 bronchoscopists were stratified into a novice group, an intermediate group, and an expert group by experience. In a randomized crossover design, each participant performed one AI-guided and one conventional (unguided) bronchoscopy procedure using a phantom. Performance was assessed by bronchial segments coverage, anatomic identification, and procedure duration. The results show that, AI guidance significantly increases segments examined in all experience levels, especially in novices.^[3,5]^ It also promotes systematic examination sequence, improves procedural completeness, and efficiency.

AI has also been effectively implemented into bronchoscopy quality control program. In a multicenter study led by Ruijin Hospital, Xi’an Chest Hospital, Shenzhen Third People’s Hospital, Heidelberg University, National University of Singapore, and other collaborating institutions, the clinical value of AI-assisted real-time guidance during electronic bronchoscopy was initially demonstrated. This AI monitoring system was trained on over 50,000 bronchial images encompassing 31 predefined anatomical sites. In clinical practice, the system provides real-time prompts during endoscopy, indicating “current location”, “already examined”, or “potential omission”. The study not only validates navigational stability and generalizability of the AI system in complex cases but also rigorously assesses its real-time quality-control performance.

## AI robotic bronchoscope

AI has further been deeply integrated into bronchoscope robot. To address the high cost and limited accessibility of bronchoscope robot, a collaborative team from the department of pulmonary and critical care medicine of Ruijin Hospital and the institute of medical robotics at Shanghai Jiao Tong University developed an AI copilot single-use flexible bronchoscope robot. The team first designed a clamp-on robotic arm that is fully compatible with single-use flexible bronchoscope. Controlled by the AI system, the arm autonomously performs advancement, rotation, flexion, and locking maneuvers. This robotic requires only replacement of a disposable single-use bronchoscope after each procedure, greatly broadening application scenarios, lowering deployment barriers, and facilitating widespread adoption in primary-care institutions. The robotic AI system collects multimodal data, including physician hand movements, electromyographic signals, and force feedback, to distinguish expert from novice techniques and generate high-fidelity learning templates. The phantom and animal validation have shown excellent operational precision and stability, paving the way for clinical adoption.

## Limitations and challenges of AI-assisted bronchoscopy

Despite the rapid technological breakthroughs in AI-assisted bronchoscopy, several critical limitations and challenges remain to be addressed.

A major issue is the limited generalizability and algorithmic robustness of current AI models. Most AI models are primarily trained on high-quality, retrospective datasets or offline videos from tertiary hospitals. In real-world clinical settings, their performance often exhibits significant deviations when confronted with variations in bronchoscopy hardware from different manufacturers, image reconstruction parameters, and real-time dynamic procedures. Furthermore, the field generally lacks robust high-level clinical evidence, such as large-scale prospective, multicenter and randomized controlled trials.

Moreover, standardized real-time quality control and supervision mechanisms remain lacking, limiting effective monitoring of image quality, navigation accuracy, and dynamic risks. Ethical issues regarding responsibility division among physicians, developers, or hospitals in AI-assisted operations or decisions, remain unclear.^[6]^ Another challenge lies in the integration of AI into the clinical workflow of bronchoscopy. Actual deployment of AI software often faces issues such as poor system compatibility and non-intuitive interfaces, causing physicians to frequently switch systems, increasing the learning costs or interrupting operational flow, thereby extending procedure time and making it difficult to meet the demands of high-intensity clinical environments.

## AI-assisted bronchoscopy: future research and outlook

In the future, AI-assisted bronchoscopy will evolve to a highly precise, real-time, and patient-specific technology. The integration of augmented reality/mixed reality (AR/MR), multimodal fusion, and advanced wearable interfaces transform bronchoscopy into a fully immersive and intelligent experience. Moreover, AI will become a genuine collaborator in dynamic lesion tracking, procedural guidance, delivering automated reports, and postoperative monitoring, which ultimately creates a seamless intelligent system spanning the entire patient journey. Overall, with the power of AI, future bronchoscopy will undergo a paradigm shift, leading respiratory disease management into a truly intelligent era.
